# 3,5,3′-Trihy­droxy-4′-meth­oxy-7-(3-methyl­but-2-en­yloxy)flavone

**DOI:** 10.1107/S1600536811005393

**Published:** 2011-02-19

**Authors:** Sheng-Hua Zhu, Shao-Qian Liu

**Affiliations:** a Guangdong Food and Drug Vocational College, Guangzhou 510520, People’s Republic of China; b Jiangxi Key Laboratory of Organic Chemistry, Jiangxi Science & Technology Normal University, Nanchang 330013, People’s Republic of China

## Abstract

The title compound pteleifolosin C, C_21_H_20_O_7_, was isolated from the petroleum ether-soluble fraction of an indigenous Chinese tree *Melicope pteleifolia* (Rutaceae). The dihedral angle between the benzene rings is 2.7 (2)°. Intra­molecular O—H⋯O hydrogen bonds occur. In the crystal, mol­ecules are linked by inter­molecular O—H—O hydrogen bonds.

## Related literature

For the medicinal usage of *M. pteleifolia* in China, see: Chinese Pharmacopoeia (1977 ▶) and for folk use of *M. pteleifolia* in South East Asia, see: Gunawardana *et al.* (1987 ▶); Shaari *et al.* (2006 ▶). For related structures and background to pteleifolosin C, see: Smith *et al.* (2001 ▶); Sultana *et al.* (1999 ▶).
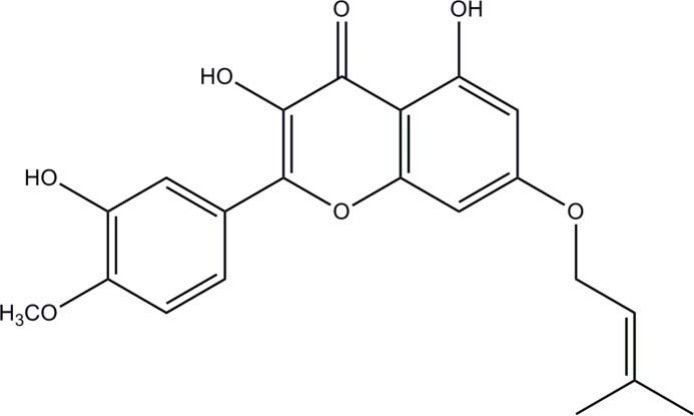


## Experimental

### 

#### Crystal data

C_21_H_20_O_7_
*M* *_r_* = 384.37
Triclinic, 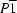

*a* = 8.4073 (18) Å
*b* = 9.0343 (19) Å
*c* = 12.489 (3) Å
α = 79.371 (2)°
β = 83.519 (3)°
γ = 78.806 (3)°
*V* = 911.7 (3) Å^3^
*Z* = 2
Mo *K*α radiation
μ = 0.11 mm^−1^
*T* = 296 K
0.60 × 0.50 × 0.45 mm

#### Data collection

Bruker APEXII CCD diffractometer
Absorption correction: multi-scan (*SADABS*; Sheldrick, 1996 ▶) *T*_min_ = 0.939, *T*_max_ = 0.954
8186 measured reflections
4103 independent reflections
3126 reflections with *I* > 2σ(*I*)
*R*_int_ = 0.019

#### Refinement

*R*[*F* ^2^ > 2σ(*F* ^2^)] = 0.046
*wR*(*F* ^2^) = 0.114
*S* = 0.98
4103 reflections
259 parameters
H-atom parameters constrained
Δρ_max_ = 0.21 e Å^−3^
Δρ_min_ = −0.22 e Å^−3^

### 

Data collection: *APEX2* (Bruker, 2004 ▶); cell refinement: *SAINT* (Bruker, 1997 ▶); data reduction: *SAINT*; program(s) used to solve structure: *SHELXS97* (Sheldrick, 2008 ▶); program(s) used to refine structure: *SHELXL97* (Sheldrick, 2008 ▶); molecular graphics: *SHELXTL* (Sheldrick, 2008 ▶); software used to prepare material for publication: *SHELXTL*.

## Supplementary Material

Crystal structure: contains datablocks I, global. DOI: 10.1107/S1600536811005393/jh2258sup1.cif

Structure factors: contains datablocks I. DOI: 10.1107/S1600536811005393/jh2258Isup2.hkl

Additional supplementary materials:  crystallographic information; 3D view; checkCIF report

## Figures and Tables

**Table 1:** Hydrogen-bond geometry (Å, °)

*D*—H⋯*A*	*D*—H	H⋯*A*	*D*⋯*A*	*D*—H⋯*A*
O5—H5⋯O4	0.82	2.21	2.6682 (17)	115
O5—H5⋯O6^i^	0.82	2.04	2.7914 (16)	153
O6—H6⋯O7	0.82	2.19	2.6440 (16)	115
O2—H2⋯O4	0.82	1.88	2.6155 (17)	148

Symmetry code: (i) 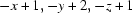.

**Fig. 1.**
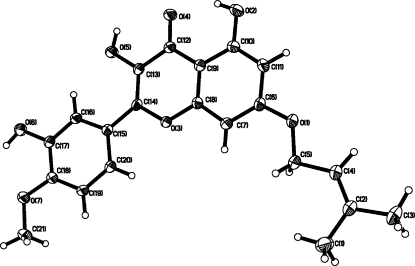
Molecular view the atom-labelling scheme. Ellipsoids are drawn at the 30% probability level. H atoms are represented as small spheres of arbitrary radii.

## supplementary crystallographic information

### Comment

The title substance is a new compound named pteleifolosin C, which is from petroleum ether soluble fraction of an indigenous Chinese tree Melicope pteleifolia, Rutaceae. In the southern area of China and in the neighboring district of South East Asia, Melicope pteleifolia is a medical herb and an edible plant as well (Gunawardana *et al.*, 1987; Shaari *et al.*, 2006). As a staple material of Guang Dong herbal tea, it also serves as a medical herb for the treatment of injury, wounds, fester and eczema (Chinese Pharmacopoeia, 1977). Nowadays it is used as a constituent in many Chinese patent medicines. In order to find its bioactive ingredients we studied the chemical composition of its leaves and found pteleifolosin C among other flavones.

### Experimental

The dried leaves powder (5 K g) of *M*. pteleifolia was percolated with 80% EtOH to yield crude extract which was fractionated in a Soxhlet to give petroleum ether, ethyl acetate, acetone and methanol soluble fraction successively. The petroleum ether fraction was subjected to column chromatography over silica gel using solvents of increasing polarity. The fraction obtained with 25% ethyl acetate in petroleum ether was subsequently subjected to gel filtration (Sephadex LH-20) eluting with CHCl_3_ and CH_3_OH (1:1) mixtures to give yellow powder, which was purified by prep. HPLC and yielded pteleifolosin C (25 mg). It is similar to the flavones found in the same genus with the O-prenylated side chain (Sultana *et al.*, 1999; Smith *et al.*, 2001). ^1^HNMR (500 MHz, DMSO-*d_6_*): δ 9.55 (1*H*, s, –OH), 12.43 (1*H*, s, –OH), 6.33 (1*H*, d, J=2.0 Hz), 6.72 (1*H*, d, J=2.0 Hz), 7.72 (1*H*, d, J=1.6 Hz), 9.31 (1*H*, s, –OH), 7.09 (1*H*, d, J=8.5 Hz), 7.68 (1*H*, d, J=1.6, 8.5 Hz), 4.64 (2*H*, d, J=6.5 Hz), 5.46 (1*H*, t, J=6.5 Hz), 1.76 (3*H*, s), 1.73 (3*H*, s), 3.85 (3*H*, s);

The dihedral angle between the benzene ring C6—C11 and the benzene ring C15—C20 is 2.7 (2)°. and the dihedral angle between the ring C8, C9, C12, C13, C14, O3 and the benzene ring C15—C20 is 2.2 (2) °. The C2—C4 (1.319 (2) Å) and C13—C14 (1.359 (2) Å) are double bands and are significantly shorter than the other C—C bond (e.g.The distance between the single band C1—C2 is 1.495 (3) Å)

### Refinement

All H atoms attached to C were fixed geometrically and treated as riding with C—H = 0.96 Å (methyl) or 0.93 Å (aromatic) with *U*_iso_(H)= 1.2*U*_eq_ (aromatic) or *U*_iso_(H) = 1.5*U*_eq_ (methyl).

### Crystal data

**Table:**

C_21_H_20_O_7_	*Z* = 2
*M**_r_* = 384.37	*F*(000) = 404
Triclinic, *P*1	*D*_x_ = 1.400 Mg m^−^^3^
*a* = 8.4073 (18) Å	Mo *K*α radiation, λ = 0.71073 Å
*b* = 9.0343 (19) Å	Cell parameters from 3191 reflections
*c* = 12.489 (3) Å	θ = 0.0–0.0°
α = 79.371 (2)°	µ = 0.11 mm^−^^1^
β = 83.519 (3)°	*T* = 296 K
γ = 78.806 (3)°	Block, colourless
*V* = 911.7 (3) Å^3^	0.60 × 0.50 × 0.45 mm

### Data collection

**Table:**

Bruker APEXII CCD diffractometer	4103 independent reflections
Radiation source: fine-focus sealed tube	3126 reflections with *I* > 2σ(*I*)
graphite	*R*_int_ = 0.019
φ and ω scans	θ_max_ = 27.5°, θ_min_ = 2.3°
Absorption correction: multi-scan (*SADABS*; Sheldrick, 1996)	*h* = −10→10
*T*_min_ = 0.939, *T*_max_ = 0.954	*k* = −11→11
8186 measured reflections	*l* = −16→16

### Refinement

**Table:**

Refinement on *F*^2^	Primary atom site location: structure-invariant direct methods
Least-squares matrix: full	Secondary atom site location: difference Fourier map
*R*[*F*^2^ > 2σ(*F*^2^)] = 0.046	Hydrogen site location: inferred from neighbouring sites
*wR*(*F*^2^) = 0.114	H-atom parameters constrained
*S* = 0.98	*w* = 1/[σ^2^(*F*_o_^2^) + (0.0437*P*)^2^ + 0.4019*P*] where *P* = (*F*_o_^2^ + 2*F*_c_^2^)/3
4103 reflections	(Δ/σ)_max_ = 0.001
259 parameters	Δρ_max_ = 0.21 e Å^−^^3^
0 restraints	Δρ_min_ = −0.22 e Å^−^^3^

### Special details

**Table:**

Geometry. All e.s.d.'s (except the e.s.d. in the dihedral angle between two l.s. planes) are estimated using the full covariance matrix. The cell e.s.d.'s are taken into account individually in the estimation of e.s.d.'s in distances, angles and torsion angles; correlations between e.s.d.'s in cell parameters are only used when they are defined by crystal symmetry. An approximate (isotropic) treatment of cell e.s.d.'s is used for estimating e.s.d.'s involving l.s. planes.
Refinement. Refinement of *F*^2^ against ALL reflections. The weighted *R*-factor *wR* and goodness of fit *S* are based on *F*^2^, conventional *R*-factors *R* are based on *F*, with *F* set to zero for negative *F*^2^. The threshold expression of *F*^2^ > σ(*F*^2^) is used only for calculating *R*-factors(gt) *etc*. and is not relevant to the choice of reflections for refinement. *R*-factors based on *F*^2^ are statistically about twice as large as those based on *F*, and *R*- factors based on ALL data will be even larger.

### Fractional atomic coordinates and isotropic or equivalent isotropic displacement parameters (Å^2^)

**Table:**

	*x*	*y*	*z*	*U*_iso_*/*U*_eq_	
C5	0.0698 (2)	0.01370 (19)	0.72012 (14)	0.0408 (4)	
H5A	0.1690	−0.0115	0.6744	0.049*	
H5B	−0.0105	0.0810	0.6751	0.049*	
C2	−0.0595 (2)	−0.21729 (19)	0.72655 (15)	0.0416 (4)	
C4	0.0075 (2)	−0.12862 (19)	0.77422 (14)	0.0428 (4)	
H4	0.0170	−0.1574	0.8490	0.051*	
C3	−0.1194 (3)	−0.3565 (2)	0.79063 (18)	0.0584 (5)	
H3A	−0.0962	−0.3684	0.8656	0.088*	
H3B	−0.0656	−0.4454	0.7606	0.088*	
H3C	−0.2347	−0.3445	0.7866	0.088*	
C1	−0.0815 (3)	−0.1880 (2)	0.60687 (17)	0.0596 (5)	
H1A	−0.0403	−0.0973	0.5729	0.089*	
H1B	−0.1951	−0.1745	0.5962	0.089*	
H1C	−0.0234	−0.2736	0.5747	0.089*	
O1	0.10023 (15)	0.08534 (13)	0.80716 (9)	0.0457 (3)	
C9	0.30637 (18)	0.47465 (17)	0.75333 (12)	0.0334 (3)	
C7	0.18620 (19)	0.29532 (17)	0.67786 (13)	0.0351 (3)	
H7	0.1540	0.2639	0.6181	0.042*	
C8	0.25584 (17)	0.42543 (17)	0.66575 (12)	0.0308 (3)	
C11	0.2147 (2)	0.2601 (2)	0.87334 (13)	0.0433 (4)	
H11	0.1990	0.2042	0.9429	0.052*	
C6	0.16667 (19)	0.21424 (18)	0.78258 (13)	0.0368 (4)	
C10	0.2850 (2)	0.38769 (19)	0.85908 (13)	0.0404 (4)	
O2	0.3353 (2)	0.43126 (16)	0.94546 (10)	0.0612 (4)	
H2	0.3772	0.5074	0.9245	0.092*	
C14	0.33864 (17)	0.63647 (16)	0.54058 (12)	0.0296 (3)	
C12	0.37894 (18)	0.60914 (17)	0.73464 (12)	0.0337 (3)	
C13	0.39232 (18)	0.68726 (17)	0.62346 (12)	0.0327 (3)	
O3	0.27254 (13)	0.50474 (12)	0.56215 (8)	0.0341 (3)	
O4	0.42931 (16)	0.66001 (14)	0.80866 (9)	0.0477 (3)	
C19	0.2782 (2)	0.69577 (18)	0.24055 (13)	0.0383 (4)	
H19	0.2381	0.6470	0.1927	0.046*	
C15	0.33922 (17)	0.70364 (17)	0.42430 (12)	0.0304 (3)	
C18	0.33616 (19)	0.83015 (18)	0.20187 (12)	0.0348 (3)	
C16	0.39856 (19)	0.83993 (17)	0.38400 (12)	0.0343 (3)	
H16	0.4396	0.8888	0.4313	0.041*	
C17	0.39621 (19)	0.90123 (17)	0.27506 (13)	0.0348 (3)	
C20	0.27958 (19)	0.63340 (18)	0.35041 (13)	0.0356 (3)	
H20	0.2400	0.5429	0.3755	0.043*	
O7	0.34083 (16)	0.90551 (14)	0.09652 (9)	0.0479 (3)	
O6	0.45551 (17)	1.03420 (14)	0.23818 (10)	0.0501 (3)	
H6	0.4399	1.0631	0.1734	0.075*	
C21	0.2862 (3)	0.8369 (2)	0.01710 (15)	0.0635 (6)	
H21A	0.3524	0.7381	0.0141	0.095*	
H21B	0.2944	0.9006	−0.0532	0.095*	
H21C	0.1749	0.8255	0.0367	0.095*	
O5	0.46006 (16)	0.81540 (14)	0.60612 (9)	0.0478 (3)	
H5	0.4836	0.8311	0.6645	0.072*	

### Atomic displacement parameters (Å^2^)

**Table:**

	*U*^11^	*U*^22^	*U*^33^	*U*^12^	*U*^13^	*U*^23^
C5	0.0505 (9)	0.0343 (9)	0.0406 (9)	−0.0176 (7)	−0.0023 (7)	−0.0040 (7)
C2	0.0397 (9)	0.0351 (9)	0.0512 (10)	−0.0108 (7)	0.0002 (7)	−0.0085 (8)
C4	0.0513 (10)	0.0358 (9)	0.0427 (9)	−0.0165 (7)	−0.0038 (7)	−0.0007 (7)
C3	0.0605 (12)	0.0405 (11)	0.0785 (14)	−0.0238 (9)	0.0020 (10)	−0.0096 (10)
C1	0.0662 (13)	0.0615 (13)	0.0584 (12)	−0.0201 (10)	−0.0063 (10)	−0.0191 (10)
O1	0.0668 (8)	0.0371 (7)	0.0382 (6)	−0.0282 (6)	−0.0063 (5)	0.0019 (5)
C9	0.0385 (8)	0.0312 (8)	0.0319 (8)	−0.0106 (6)	−0.0025 (6)	−0.0043 (6)
C7	0.0423 (8)	0.0333 (8)	0.0335 (8)	−0.0147 (7)	−0.0058 (6)	−0.0048 (6)
C8	0.0346 (7)	0.0284 (8)	0.0297 (7)	−0.0097 (6)	−0.0020 (6)	−0.0015 (6)
C11	0.0612 (11)	0.0411 (10)	0.0297 (8)	−0.0210 (8)	−0.0037 (7)	0.0015 (7)
C6	0.0427 (9)	0.0304 (8)	0.0386 (9)	−0.0149 (7)	−0.0032 (7)	−0.0006 (7)
C10	0.0543 (10)	0.0397 (9)	0.0311 (8)	−0.0171 (8)	−0.0049 (7)	−0.0054 (7)
O2	0.1038 (11)	0.0607 (9)	0.0313 (6)	−0.0451 (8)	−0.0121 (7)	−0.0029 (6)
C14	0.0325 (7)	0.0243 (7)	0.0332 (8)	−0.0095 (6)	−0.0021 (6)	−0.0034 (6)
C12	0.0394 (8)	0.0322 (8)	0.0326 (8)	−0.0116 (6)	−0.0038 (6)	−0.0070 (6)
C13	0.0376 (8)	0.0279 (8)	0.0350 (8)	−0.0132 (6)	−0.0029 (6)	−0.0040 (6)
O3	0.0468 (6)	0.0302 (6)	0.0292 (5)	−0.0178 (5)	−0.0057 (4)	−0.0016 (4)
O4	0.0714 (8)	0.0457 (7)	0.0350 (6)	−0.0294 (6)	−0.0104 (6)	−0.0065 (5)
C19	0.0528 (10)	0.0331 (8)	0.0344 (8)	−0.0169 (7)	−0.0096 (7)	−0.0060 (7)
C15	0.0322 (7)	0.0280 (8)	0.0316 (7)	−0.0068 (6)	−0.0032 (6)	−0.0045 (6)
C18	0.0436 (8)	0.0326 (8)	0.0283 (8)	−0.0092 (7)	−0.0039 (6)	−0.0025 (6)
C16	0.0432 (8)	0.0319 (8)	0.0320 (8)	−0.0147 (6)	−0.0046 (6)	−0.0065 (6)
C17	0.0418 (8)	0.0279 (8)	0.0367 (8)	−0.0137 (6)	−0.0026 (6)	−0.0031 (6)
C20	0.0451 (9)	0.0290 (8)	0.0357 (8)	−0.0151 (7)	−0.0054 (7)	−0.0029 (6)
O7	0.0756 (9)	0.0419 (7)	0.0299 (6)	−0.0223 (6)	−0.0089 (6)	0.0000 (5)
O6	0.0808 (9)	0.0405 (7)	0.0363 (6)	−0.0356 (6)	−0.0079 (6)	0.0029 (5)
C21	0.1061 (17)	0.0570 (13)	0.0328 (9)	−0.0242 (12)	−0.0198 (10)	−0.0021 (9)
O5	0.0753 (8)	0.0424 (7)	0.0364 (6)	−0.0362 (6)	−0.0113 (6)	−0.0028 (5)

### Geometric parameters (Å, °)

**Table:**

C5—O1	1.4328 (19)	C10—O2	1.3501 (19)
C5—C4	1.498 (2)	O2—H2	0.8200
C5—H5A	0.9700	C14—C13	1.359 (2)
C5—H5B	0.9700	C14—O3	1.3793 (16)
C2—C4	1.319 (2)	C14—C15	1.467 (2)
C2—C1	1.495 (3)	C12—O4	1.2513 (17)
C2—C3	1.503 (2)	C12—C13	1.440 (2)
C4—H4	0.9300	C13—O5	1.3590 (17)
C3—H3A	0.9600	C19—C18	1.380 (2)
C3—H3B	0.9600	C19—C20	1.384 (2)
C3—H3C	0.9600	C19—H19	0.9300
C1—H1A	0.9600	C15—C20	1.396 (2)
C1—H1B	0.9600	C15—C16	1.404 (2)
C1—H1C	0.9600	C18—O7	1.3655 (19)
O1—C6	1.3581 (18)	C18—C17	1.396 (2)
C9—C8	1.390 (2)	C16—C17	1.372 (2)
C9—C10	1.419 (2)	C16—H16	0.9300
C9—C12	1.433 (2)	C17—O6	1.3713 (18)
C7—C6	1.385 (2)	C20—H20	0.9300
C7—C8	1.389 (2)	O7—C21	1.421 (2)
C7—H7	0.9300	O6—H6	0.8200
C8—O3	1.3650 (18)	C21—H21A	0.9600
C11—C10	1.369 (2)	C21—H21B	0.9600
C11—C6	1.400 (2)	C21—H21C	0.9600
C11—H11	0.9300	O5—H5	0.8200
			
O1—C5—C4	105.77 (13)	O2—C10—C9	119.45 (14)
O1—C5—H5A	110.6	C11—C10—C9	120.28 (14)
C4—C5—H5A	110.6	C10—O2—H2	109.5
O1—C5—H5B	110.6	C13—C14—O3	119.66 (13)
C4—C5—H5B	110.6	C13—C14—C15	128.81 (13)
H5A—C5—H5B	108.7	O3—C14—C15	111.53 (12)
C4—C2—C1	123.26 (17)	O4—C12—C9	123.70 (14)
C4—C2—C3	121.40 (17)	O4—C12—C13	119.81 (14)
C1—C2—C3	115.33 (16)	C9—C12—C13	116.49 (13)
C2—C4—C5	126.59 (16)	C14—C13—O5	121.85 (14)
C2—C4—H4	116.7	C14—C13—C12	121.80 (13)
C5—C4—H4	116.7	O5—C13—C12	116.35 (13)
C2—C3—H3A	109.5	C8—O3—C14	121.51 (11)
C2—C3—H3B	109.5	C18—C19—C20	120.15 (14)
H3A—C3—H3B	109.5	C18—C19—H19	119.9
C2—C3—H3C	109.5	C20—C19—H19	119.9
H3A—C3—H3C	109.5	C20—C15—C16	118.06 (14)
H3B—C3—H3C	109.5	C20—C15—C14	120.56 (13)
C2—C1—H1A	109.5	C16—C15—C14	121.38 (13)
C2—C1—H1B	109.5	O7—C18—C19	126.65 (13)
H1A—C1—H1B	109.5	O7—C18—C17	114.35 (14)
C2—C1—H1C	109.5	C19—C18—C17	118.99 (14)
H1A—C1—H1C	109.5	C17—C16—C15	120.24 (13)
H1B—C1—H1C	109.5	C17—C16—H16	119.9
C6—O1—C5	119.15 (12)	C15—C16—H16	119.9
C8—C9—C10	118.12 (14)	C16—C17—O6	118.83 (13)
C8—C9—C12	119.70 (14)	C16—C17—C18	121.24 (14)
C10—C9—C12	122.18 (14)	O6—C17—C18	119.93 (14)
C6—C7—C8	117.30 (14)	C19—C20—C15	121.31 (14)
C6—C7—H7	121.3	C19—C20—H20	119.3
C8—C7—H7	121.3	C15—C20—H20	119.3
O3—C8—C7	116.47 (13)	C18—O7—C21	117.28 (13)
O3—C8—C9	120.82 (13)	C17—O6—H6	109.5
C7—C8—C9	122.71 (14)	O7—C21—H21A	109.5
C10—C11—C6	119.59 (15)	O7—C21—H21B	109.5
C10—C11—H11	120.2	H21A—C21—H21B	109.5
C6—C11—H11	120.2	O7—C21—H21C	109.5
O1—C6—C7	124.01 (14)	H21A—C21—H21C	109.5
O1—C6—C11	114.00 (14)	H21B—C21—H21C	109.5
C7—C6—C11	121.99 (14)	C13—O5—H5	109.5
O2—C10—C11	120.26 (15)		
			
C1—C2—C4—C5	0.8 (3)	C15—C14—C13—C12	178.36 (14)
C3—C2—C4—C5	−179.14 (17)	O4—C12—C13—C14	−179.72 (15)
O1—C5—C4—C2	168.75 (17)	C9—C12—C13—C14	0.2 (2)
C4—C5—O1—C6	176.45 (14)	O4—C12—C13—O5	−0.4 (2)
C6—C7—C8—O3	−179.33 (14)	C9—C12—C13—O5	179.51 (14)
C6—C7—C8—C9	0.5 (2)	C7—C8—O3—C14	179.37 (13)
C10—C9—C8—O3	179.69 (14)	C9—C8—O3—C14	−0.5 (2)
C12—C9—C8—O3	−0.8 (2)	C13—C14—O3—C8	1.6 (2)
C10—C9—C8—C7	−0.2 (2)	C15—C14—O3—C8	−178.21 (12)
C12—C9—C8—C7	179.31 (14)	C13—C14—C15—C20	179.39 (15)
C5—O1—C6—C7	9.8 (2)	O3—C14—C15—C20	−0.8 (2)
C5—O1—C6—C11	−170.35 (15)	C13—C14—C15—C16	−0.9 (2)
C8—C7—C6—O1	179.81 (14)	O3—C14—C15—C16	178.91 (13)
C8—C7—C6—C11	−0.1 (2)	C20—C19—C18—O7	−179.26 (16)
C10—C11—C6—O1	179.35 (15)	C20—C19—C18—C17	0.2 (2)
C10—C11—C6—C7	−0.8 (3)	C20—C15—C16—C17	0.4 (2)
C6—C11—C10—O2	−178.46 (17)	C14—C15—C16—C17	−179.33 (14)
C6—C11—C10—C9	1.1 (3)	C15—C16—C17—O6	−179.80 (14)
C8—C9—C10—O2	178.91 (15)	C15—C16—C17—C18	−0.3 (2)
C12—C9—C10—O2	−0.5 (3)	O7—C18—C17—C16	179.55 (15)
C8—C9—C10—C11	−0.7 (2)	C19—C18—C17—C16	0.0 (2)
C12—C9—C10—C11	179.86 (16)	O7—C18—C17—O6	−1.0 (2)
C8—C9—C12—O4	−179.14 (15)	C19—C18—C17—O6	179.51 (15)
C10—C9—C12—O4	0.3 (3)	C18—C19—C20—C15	−0.1 (3)
C8—C9—C12—C13	1.0 (2)	C16—C15—C20—C19	−0.2 (2)
C10—C9—C12—C13	−179.57 (15)	C14—C15—C20—C19	179.55 (15)
O3—C14—C13—O5	179.23 (13)	C19—C18—O7—C21	−2.5 (3)
C15—C14—C13—O5	−1.0 (3)	C17—C18—O7—C21	178.09 (16)
O3—C14—C13—C12	−1.5 (2)		

### Hydrogen-bond geometry (Å, °)

**Table:**

*D*—H···*A*	*D*—H	H···*A*	*D*···*A*	*D*—H···*A*
O5—H5···O4	0.82	2.21	2.6682 (17)	115
O5—H5···O6^i^	0.82	2.04	2.7914 (16)	153
O6—H6···O7	0.82	2.19	2.6440 (16)	115
O2—H2···O4	0.82	1.88	2.6155 (17)	148

Symmetry codes: (i) −*x*+1, −*y*+2, −*z*+1.
